# Best of Both Worlds: Detecting Application Layer Attacks through 802.11 and Non-802.11 Features

**DOI:** 10.3390/s22155633

**Published:** 2022-07-28

**Authors:** Efstratios Chatzoglou, Georgios Kambourakis, Christos Smiliotopoulos, Constantinos Kolias

**Affiliations:** 1 Department of Information & Communication Systems Engineering, University of the Aegean, 83200 Karlovasi, Greece; efchatzoglou@gmail.com (E.C.); csmiliotopoulos@aegean.gr (C.S.); 2 Joint Research Centre, European Commission, 21027 Ispra, Italy; 3 Department of Computer Science, University of Idaho, Idaho Falls, ID 83402, USA; kolias@uidaho.edu

**Keywords:** intrusion detection systems, 802.11, Wi-Fi, network security, machine learning

## Abstract

Intrusion detection in wireless and, more specifically, Wi-Fi networks is lately increasingly under the spotlight of the research community. However, the literature currently lacks a comprehensive assessment of the potential to detect application layer attacks based on both 802.11 and non-802.11 network protocol features. The investigation of this capacity is of paramount importance since Wi-Fi domains are often used as a stepping stone by threat actors for unleashing an ample variety of application layer assaults. In this setting, by exploiting the contemporary AWID3 benchmark dataset along with both shallow and deep learning machine learning techniques, this work attempts to provide concrete answers to a dyad of principal matters. First, what is the competence of 802.11-specific and non-802.11 features when used separately and in tandem in detecting application layer attacks, say, website spoofing? Second, which network protocol features are the most informative to the machine learning model for detecting application layer attacks? Without relying on any optimization or dimensionality reduction technique, our experiments, indicatively exploiting an engineered feature, demonstrate a detection performance up to 96.7% in terms of the Area under the ROC Curve (AUC) metric.

## 1. Introduction

Data traffic over wireless networks is exhibiting ever-increasing growth. Due to its ability to offer increased mobility, speed, usability, and low installation and maintenance costs, IEEE 802.11 networks are at the epicenter of this rapid shift to a wireless realm. Such networks, commercially known as Wi-Fi, are omnipresent in our daily life for providing connectivity to areas facilitating a wide spectrum of contemporary services [1], including Voice over Wi-Fi (VoWiFi) and automotive and smart city applications.

On the other hand, mainstream digital technologies are also in the crosshairs of a variety of threat actors. Furthermore, while the 802.11 standard has greatly advanced over the years in terms of security, recent research work indicates that even the latest defenses, say, the Simultaneous Authentication of Equals (SAE) authentication and key exchange method and the Protected Management Frames (PMF) mechanism, embraced by the most recent at the time of writing 802.11-2020 standard are not impermeable [2,3,4]. Through a security prism, the situation becomes more cumbersome and complicated, given that at least infrastructure-based Wi-Fi domains co-exist with their wired counterparts, and therefore the former can be used as a springboard for attacking the latter.

In this context, Intrusion Detection Systems (IDS) provide a supplementary layer of defense either to purely wireless domains or others that exploit a mixture of wired and wireless zones, trusted, say, within the premises of an enterprise, or not. Thus, far, a significant mass of works has investigated Machine Learning (ML) driven IDS both for wireless and wired networks and through the lens of diverse benchmark datasets and techniques. However, most likely due to the lack of proper datasets, research on IDS capitalizing simultaneously on 802.11-oriented and other types of network protocol features, including TCP, UDP, and Address Resolution Protocol (ARP), strikingly lags behind.

*Our contribution:* The work at hand aspires to fill this important literature gap by exploiting the modern AWID3 benchmark dataset. AWID3 contains a rich repertoire of attacks, which span from legacy 802.11 ones, say, deauthentication, to application layer assaults, including amplification, malware, botnet, SQL injection, and others. This renders AWID3 an ideal testing platform for assessing IDS that target the detection of a wide variety of attacks mounted on diverse layers of the protocol stack. Under this angle, and by considering an opponent who takes advantage of a Wi-Fi domain to launch application layer attacks, the current work answers the following key questions, which to the best of our knowledge are neglected by the related work:

- Given two different network protocol feature sets, the first comprising 802.11-specific features and the second encompassing an assortment of non-802.11 features, which of them is superior in detecting application layer attacks, and to what degree? The features included in each feature are selected based on prior work on the topic.
- Which features per set are the most important and bear the most information to the ML model?
- How the IDS detection performance is affected if the two above-mentioned feature sets are combined and possibly escorted by engineered (artificial) features? Note that an engineered feature aims at improving the detection of a cumbersome identify class of attacks.

To respond to these questions, we performed a series of experiments utilizing both shallow and deep learning techniques. It is important to note that, in the context of the current work, the term “non-802.11” does not embrace any application layer feature. This makes the responses to the above questions more interesting, given that, typically, the detection of application layer attacks involves features of the same layer, which, however, are not normally available due to encryption or anonymization.

The rest of the manuscript is divided into sections as follows. The next section presents the related work on the topic. Section 3 details the feature selection and data preprocessing schemes. The results after experimenting with each set of features are included in Section 4. A deeper look into feature importance is provided in Section 5. The same section offers an additional set of experiments performed over a unified feature set, and elaborates on the potential of engineered features. The last section provides concluding remarks and describes future research avenues.

## 2. Related Work

The current section briefly reviews the relevant literature. We consider major contributions spanning a time period from 2011 to 2021. The section only embraces works focusing on the identification of layer attacks through ML techniques utilizing non-802.11 network protocol features; in this respect, we do not consider works that deal with application layer attacks in general [5,6,7]. The emphasis is put on the feature selection process, the utilized methodology, and the ML algorithm or models utilized per work. The reader should keep in mind that this section purposefully omits related work examining Wireless IDS (WIDS) capitalizing on 802.11-specific features. For such contributions, the reader is referred to [8].

In [9], the authors relied on a three-layered Neural Network (NN) structure to perform IoT network traffic flow classification within the context of a proposed IDS. Both binary and multiclass classification via a Feedforward NN (FNN) model were conducted against the *Bot-IoT* dataset [10], and towards the identification of 10 diverse classes of IoT-oriented attacks. The layers of the FNN model were randomly weighted based on a sampled version of the initial normal data distribution. The performance of the model was evaluated through legacy metrics, including Accuracy and F1. Twenty-five high-level features were selected from the dataset, representing diverse related field categories pertaining to different traffic, including ARP, IP, TCP, and UDP. Specifically, FNN achieved an F1 score above 99% in both the classification categories, i.e., binary and multiclass. The results were compared against the Support Vector classifier model with 5-fold cross-validation, achieving 82% at best. On the downside, the proposed IDS failed to generalize the classification procedure presenting low precision during the identification of specific categories of attacks or even variations of the same attack, namely flooding and reconnaissance ones, for both binary and multiclass experiments.

The authors in [11] proposed a DDoS IDS for the classification of malicious traffic with the Gradient Boosting (GBT) algorithm. The experiments utilized two custom datasets created from the real-world Internet traffic traces dataset obtained from *CAIDA* [12]. The proposed scheme was evaluated against GBT algorithms metrics, achieving an F1 score above 95% with a False Positive Rate (FPR) between 9% and 12%, especially when large iteration and DT values were applied. However, the authors provide minimal details regarding the creation of the two datasets and the feature selection procedure.

The authors in [13,14] concentrated on the identification and categorization of encrypted traffic using Skype and SSH as case studies. The IP packet header along with flow-based features was extracted from various public and private datasets, including *DARPA-99* and *NIMS*. They evaluated their proposal against various classifiers and DNN models in the context of binary and multiclass classification. The authors chose 61 basic features. By capitalizing on them, they also constructed a set of UDP and TCP flow-based (artificial) features, without, however, properly justifying their choices, and the way these engineered features were utilized in the context of the NN models.

The work in [15] relied on a supervised ML approach to develop a dual-layer IoT IDS, which is destined for malicious traffic classification and subsequently aids in differentiating between attack types. They executed five attacks, namely network scanning, DoS, evil twin, Man in the Middle (MiTM), and injection, on a custom-made IoT testbed and created a dataset comprising 88 features. Following a feature selection process, they resulted in two subsets of 29 and 9 features. The experiments carried out with a handful of ML models, namely Multinomial Naive Bayes (MNB), Support Vector Machines (SVM), Decision Trees (DT), Random Forest (RF), and Artificial Neural Networks (ANN), achieved a score above 95% and 92% regarding the F1 metric for malicious traffic and attack recognition models, respectively. During the preprocessing phase of the dataset, all the missing values were replaced with a zero value, possibly raising the risk of affecting or misleading the performance and effectiveness of the models in such an imbalanced dataset.

Several other contributions were dedicated to the classification of higher-layer attacks with ML techniques. The authors in [16] relied on C4.5 decision tree and Symbiotic Bid-based (SBB) Genetic Programming (GP) models for the creation of a botnet classification mechanism. The work in [17] implemented two DNN models, namely, Autoencoder and CNN, to perform feature selection and classification of TLS traffic. Moreover, the author in [18] put forward a hybrid KNN-GP classification approach for the identification of DDoS traffic. Despite the promising results, the three aforementioned papers suggest a feature implementation approach that relies on custom extracted flow-based statistical measurements, providing little information regarding their extraction process. Nevertheless, an approach that totally neglects features based on header fields may lead to dubious results. Precisely, engineered features are interlinked with a specific attack, and a slight deviation in the underlying settings may cause the model to fail to generalize to even minor variations of the same attack.

The authors in [19] relied on DNN models to assess the performance of an IDS protecting against DDoS attacks. For model training, the authors implemented the extended and imbalanced “UNB ISCX Intrusion Detection Evaluation 2012 DataSet”. The evaluation of the models was conducted across several NN models, namely CNN, RNN, LSTM, and GRU. The authors created a balanced dataset that was sampled repeatedly prior to the execution of each classification model’s experiment. It can be said that the continuous re-sampling of the original dataset along with data normalization should be executed as a preprocessing step in conjunction with feature importance for avoiding compromising the integrity of the final results and the overall generalization of the created model.

In [20], the authors proposed two feature selection algorithms, namely *Chi Square* and *Symmetrical*, together with *Decision Tree* to effectively identify and detect DDoS assaults. They took advantage of five different subsets stemming from the CAIDA [12] dataset. Their experiments revealed that from the 25 initially selected features, only seven contributed to positively achieving a precision score above 95%. The authors do not elaborate on whether and in which way the feature selection process conducted on the CAIDA subsets can influence the effectiveness of a generalized IDS.

The authors in [21] proposed a dataset, namely *Edge-IIoTset*, destined to IoT and Industrial IoT (IIoT) applications. As an initial step, they collected network traffic from a great variety of IoT devices and digital sensors during the execution of 14 IoT-related attacks, which were derived from five generalized categories: information gathering, DDoS, MiTM, injection, and malware. Nearly 1.2K features were identified, from which only the 61 most relevant were finally selected. Categorical data conversion into ML algorithm’s compatible form was carried out by means of the *pandas.get_dummies* Python library, while duplicate and missing values, including “NAN” and “INF”, were removed. Above that, flow-based features related to IP addresses, port, payload information, and timestamps were dropped as irrelevant to the concept of the proposed dataset. Both supervised swallow classification and DNN analysis were utilized to evaluate the effectiveness of the proposed IDS model. The authors relied on hyperparameter tuning using Grid Search, tying their ML models exclusively to the proposed data set. We argue that the aforesaid approach does not highlight the general nature of the conducted experiments and emphasizes how the proposed analysis may apply to unknown data beyond the presented work.

The work in [22] introduced an FNN-based IDS for multiclass classification of high-layer attacks on IoT devices. Regularization and hyperparameter model tuning was adopted by the authors, while the final results of the FNN model were compared against the linear-SVM supervised algorithm. They concluded that FNN is more time efficient and expands better vis-à-vis the SVM model. The authors relied on frame-, ARP-, TCP-, IP-, and UDP-related fields during the feature extraction procedure. However, the absence of feature importance verification in the selected fields could not corroborate the robustness of the 29 selected features.

The authors in [23] presented another dataset, coined *UKM-IDS20*, comprising 46 features extracted from DoS, ARP poisoning, network scanning, and malware attack traffic. The dataset was evaluated through Artificial NN against the legacy *KDD99* and *UNSW-NB15* datasets, revealing higher attack detection rates. It can be said that, as a rule of thumb, engineered features may be tightly interrelated to the described testbed scenarios, and therefore even tiny variations of an attack may go undetected.

The contributions in [24,25] coped with unsupervised DNN techniques towards the creation of IDS specially designed to identify higher-layer attacks. Precisely, the authors in [24] assessed two datasets comprising EtherNet/IP and Modbus protocol packets. Stacked denoising autoencoders NN were used to train and evaluate the proposed IDS. Above that, the work in [25] implemented a signature-based ML approach, dubbed “Classification Voting”, in an effort to deliver a packet-agnostic IDS. Both these approaches provide little information regarding the feature selection procedure.

Works such as [26,27,28] are considered marginally within the scope of the current paper as they focus on the comparative presentation of commonly used classifiers, NN models, and feature selection techniques towards the creation of an IDS. Moreover, the authors in [29] presented an adversarial approach that is applicable to the falsification concept of LSTM-based IDS targeting DDoS traffic. This survey is also considered marginally relevant to ours as it examines the manipulation of high-layer features towards bypassing DDoS detection.

To ease the parsing of the relevant literature, Table 1 summarizes the pertinent characteristics of each work included in this section. Namely, we outline the features selected per work plus the classification methods used. It is important to point out that the non-802.11 features shown in boldface in Table 1 are common to that listed in the penultimate column of Table 2, i.e., the features used in the context of this work. Overall, most of the works discussed in this section resorted to some sort of feature selection towards the identification of malicious traffic [9,11,13,15,16,17,18,19,20,21,22,23,24,25,29]. To this end, the majority of contributions implemented binary or multiclass classification with traditional algorithms such as Adaboost, KNN, C4.5, Random Forest, and Decision Trees [9,11,13,15,16,17,18,20]. Deep Learning techniques were also implemented in several cases [9,14,15,17,19,21,22,23,24,25,29,29].

Altogether, the analysis of the related work carried out in the current section alongside the argumentation provided in § 2 of [8], suggests that there is a noticeable lack of contributions attempting to detect higher-layer attacks, e.g., HTTP-oriented, by merely capitalizing on non-application features of diverse kinds.

## 3. Feature Selection and Data Preprocessing

The pertinent to this work, feature selection and data preprocessing procedures are explained in the current section and summarized in Table 2. The analysis relies on AWID3, which to our knowledge is currently the only benchmark dataset that along with 802.11-specific attacks contains several others exercised at the layer. The attacks were recorded in a WPA2-Enterprise environment with Protected Management Frames (PMF) enabled. Precisely, AWID3 includes 21 assaults ranging from legacy deauthentication to more advanced and higher-layer ones, including KRACK, amplification, malware, and botnet. It is offered in both Comma-Separated Values (CSV) (254 features) and pcap (raw data) formats. Naturally, for the purposes of this work, we concentrated only on attacks mounted on the application layer, that is, the six pcap files named *Botnet, Malware, SSH, SQL Injection, SSDP amplification*, and *Website spoofing*; for a detailed description of these attacks, the reader is referred to § 2 of [2]. The resulting dataset comprises a total of 13,645,068 or ≈50% of the entire dataset samples.

### 3.1. Feature Selection

As already emphasized, the current work attempts to rely on a dual set of features, that is, 802.11-specific and others (non-802.11), with the aim to detect application layer attacks. Therefore, the feature selection criteria differ depending on the feature set. For the 802.11-specific features, the process was straightforward, picking all but three of those justified in § 2 of  [8]. Precisely, the *radiotap.channel.freq*, *radiotap.channel.type.cck*, and *radiotap.channel.type.ofdm* were dropped from the original feature set in [8] due to not bearing any useful information in assisting the detection of application layer attacks. Put simply, all these features carry the same value across all the samples, e.g., the *radiotap.channel.type.cck* is always equal to “1”. We did include however the *wlan_radio.duration*, *wlan_radio.signal_dbm*, and *wlan_radio.phy* mentioned in § 4.3 of [8] as they are specific to AWID3 and carry useful information that can assist in the detection of layer attacks. On the other hand, the set of non-802.11 features comprised 17 ARP, IP, TCP, and UDP features commonly used in the previous work as detailed in Section 2 and in Table 1.

As already pointed out and observed from the rightmost part of Table 2, no application layer feature, say, DNS, HTTP, or other was used. This may seem unusual, given that this work does concentrate on the detection of application layer attacks. The basic reason behind this choice is that, typically, the application features are encrypted (and thus not available) due to, say, a TLS tunnel [30] or other mechanisms [31], including encrypted DNS [32]. Additionally, in certain cases, e.g., SSH, the traffic cannot be decrypted. Further, even in situations where the network traffic is either in plaintext or can be somehow decrypted, application features may need to be anonymized (obfuscated) for preserving end-users’ privacy. In this respect, the current work investigates the intriguing potential of detecting application layer attacks through readily available features; note that all the 802.11 features in the leftmost part of Table 2 are always plaintext.

Generally, in addition to the above remarks, feature selection was performed contingent on the following conditions:

- Each frame- or packet-level feature must be independent of the settings the attack was carried out, thus having zero indicators of pinpointing a specific device. Precisely, regardless of the observed values a field has in the examined dataset, it may contribute minimally to intrusion detection because there exist trivial means of spoofing these values. This for instance stands true for the *ip.src* and *ip.dst* fields, which refer to the source and destination IP address of the two communicating ends. Namely, if the attacker always operates on a specific source IP address, then the ML model will apparently learn to flag any frame stemming from this address as malicious.
- For the selection of the non-802.11 features, we relied either on the related literature (how often a feature is exploited in similar analyses with reference to Table 1) or on empirical observations (which is the actual information a feature carries for the detection of the specific classes of attacks). Overall, we resulted with 17 features, where 14 of them exit in Table 1 and the rest, namely *arp.proto.size*, *arp.hw.type*, and *arp* were empirically cherry-picked. Moreover, all the non-802.11 features are unrelated to the underlying application, say, HTTP, FTP, or DNS.
- Both sets should comprise an approximately equal number of features; having an overly greater number of features in one set might bias the IDS towards the information provided by the most populous set.
- Each selected feature is neither time-series nor flow-series related. Put simply, the feature values should be time-independent of each other, meaning that the value contained in a frame or packet is unrelated to those existing in the previous and next frames or packets.

Based on the above-mentioned criteria, as recapitulated in Table 2, 16 and 17 features have been selected for each feature set, respectively.

### 3.2. Data Preprocessing

Data preprocessing refers to the encoding, normalization, and scaling techniques used for preparing the data. As indicated in Table 2, each feature type was processed through a different conversion technique, either *Min-Max* (for features with discrete numeric values) or *One-Hot Encoding* (OHE) (for those represented by discrete values). Note that the dataset was analyzed “as is”, without changing its imbalanced nature, e.g., through a sampling technique. After that, each CSV file was searched for undefinable values, say, “Null”, “NaN”, and decimal values which end with “e-”, and the corresponding rows—around 0.02% records—were removed. Moreover, values containing a hyphen were grouped into one value; for instance, any “0-0” was changed to “0”. Empty cells were replaced with the 0 value.

Three classes were defined having the following labels: *Normal*, *Flooding*, and *Other*. The two latter classes match the six application layer attacks available in the dataset. Namely, the *Flooding* class represents SSDP amplification, website spoofing, and SSH, while the *Other* class represents botnets, malware, and SQL injection.

## 4. Experiments

As stated in Section 3.2, we did the fewest possible alterations to both the feature sets in an effort to achieve IDS generalization. We adopted commonly accepted ML techniques, without applying optimization or dimensionality reduction techniques. With reference to the methodology, the following items are noteworthy.

- For the sake of generalization, all the ML algorithms or models were common for both sets of features across all the experiments for the same type of analysis.
- The ML algorithms were chosen mainly based on the reproducibility criterion; the implementation of each selected algorithm is freely available in renowned ML libraries.
- Given that the dataset was imbalanced, we used the stratified k-fold validation method with the *k* parameter equal to 10; every k-fold test set will receive the same number of samples from each class of the dataset. Each fold for the 802.11 features was composed of 11,850,183 and 1,316,687 samples for the training and testing sets, respectively. For the non-802.11 feature test, the corresponding numbers were 12,280,559 and 1,364,507, respectively.
- For DNN analysis, a part of each fold (20%) or ≈2,370,036 and ≈2,456,111 samples were used as a validation test for 802.11 and non-802.11 feature sets, respectively. This portion of the samples was removed from the training set of each fold. As a result, each fold in DNN analysis comprised fewer samples vis-à-vis those used in swallow analysis. Recall that the validation test can aid in avoiding overfitting.
- The main goal was to prevent overfitting and augment the generalization effect. To this end, we exploited hyperparameter optimization methods, including *Grid search*, for attaining the best possible results per shallow classifier.

### 4.1. Shallow Classifiers

Classification of both sets of features was conducted against three ML models, namely decision trees (DT), LightGBM, and bagging. The experiments were conducted on an MS Windows 10 Pro AMD Ryzen 7 2700 CPU machine with 64GB RAM, without the use of a GPU. Table 3 includes the utilized parameters per classifier. The DT and bagging classifiers were coded using the *scikit-learn* v1.0.1 Python library, while LightGBM was implemented with the homonymous Python module in v.3.3.2.

Table 4 groups the shallow classification results on the two sets of features. The results represent the average score calculated over all the folds. Specifically, the table contains the most relevant evaluation metrics per classifier, namely AUC, precision (Prec), recall, F1-Score, and accuracy (Acc), along with the total time of each model’s execution in hours:min:sec format. Given the imbalanced nature of the dataset, the Acc column is included just for reasons of completeness and displayed in gray background. The best case in terms of AUC and F1 scores is shown in green text, whereas the worst case is in orange. Overall, in terms of AUC, the best performer was the bagging model, producing a 90.77% and 76.28% score for the 802.11 and non-802.11 sets of features, respectively.

**Figure 1:**
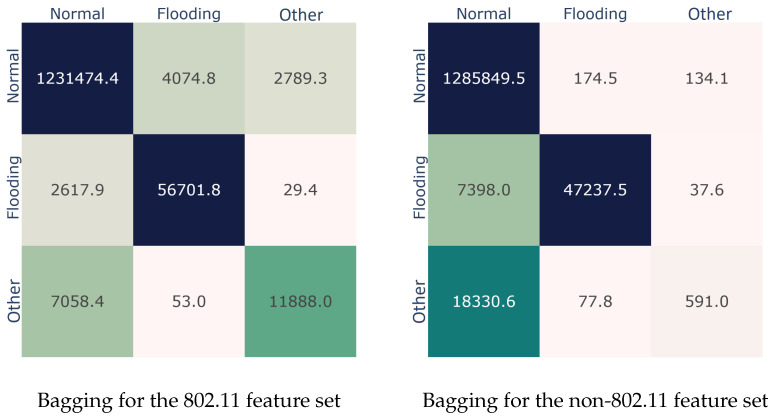
Best performer (Bagging) confusion matrices per feature set.

Figure 1 complements the above-mentioned results by depicting the confusion matrices for the best performer for both feature sets; the numbers represent the average value. Clearly, for the 802.11 feature set, the top performer demonstrated an increased AUC score due to equally unclassified samples per class. On the other hand, the non-802.11 feature set presented optimal results with the *Normal* class, only misclassifying around 300 samples. On the negative side, it missed 13.5% samples of the *Flooding* class and completely missed the *Other* class.

### 4.2. Deep Neural Networks

Regarding DNN analysis, we relied on two different well-known models, namely, Multi-Layer Perceptron (MLP) and Denoising stacked Autoencoders (AE). The experiments were performed on an MS Windows 10 Pro AMD Ryzen 7 2700 CPU machine with 64 GB RAM and a GTX 1060 6 GB GPU. Additionally, the *sklearn* v.1.0.1, *Keras* v.2.8.0, and *TensorFlow* v.2.8.0-dev20211113 in Python v3.8.10 were utilized. To hasten the training process, a GPU was used along with CUDA v11.0.

Table 5 recaps the parameters used per the DNN model. For overseeing the training phase, the mini-batch Stochastic Gradient Descent (SGD) optimizer was implemented, with a learning rate of 0.01 and a momentum of 0.9. A low *Batch* size, e.g., 150, can result in a more generalized DNN model since more data will be analyzed during each *Epoch*. To this end, a *Batch* size of 170 was used. Moreover, we exploited the well-known *ReLU* activator, where applicable. Another customary activator function for the output layer of DNN is the so-called *Softmax*. The latter was implemented to classify the results. Last but not least, the *Dropout* technique was used for the sake of adding a regularization effect.

For both the MLP and AE, the input layer was different per dataset, i.e., 43 and 58 columns for the 802.11 and non-802.11 feature sets, respectively. The output was the three classes mentioned in Section 3, namely *Normal*, *Flooding*, and *Other*.

The *Model Checkpoint* and *Early Stopping* techniques were utilized to retain the optimal training state of each DNN model. Regarding these techniques, we oversaw the minimum loss value, and in case the loss value did not refine for two successive epochs, the training phase was stopped, and the model was retrained with the last optimal epoch. Therefore, each fold was trained for no less than two more epochs. No less important, the *Dropout* and *validation test* techniques were also exploited in an effort to avoid overfitting.

**Figure 2:**
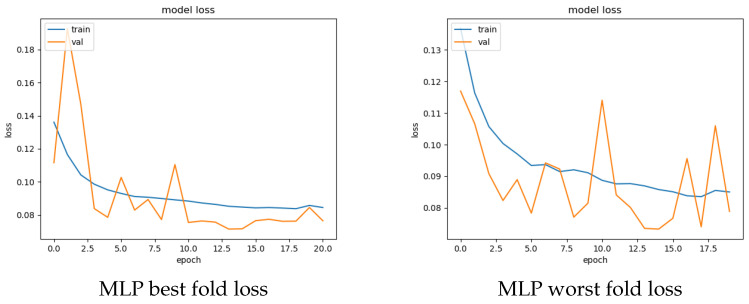
Best performer (MLP) loss per epoch performance for the 802.11 feature set.

**Figure 3:**
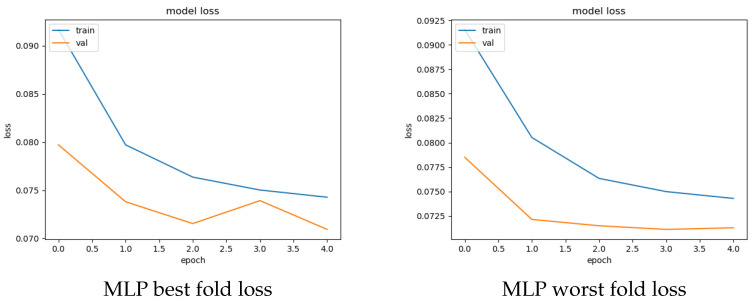
Best performer (MLP) loss per epoch performance for the non-802.11 feature set.

For both the feature sets, the average score calculated over all the folds for each examined model is given in Table 6. The table also contains the number of epochs required for training each model. As observed from the table, both models yielded similar results on both feature sets. Precisely, AE presented a very close or identical detection performance to MLP, i.e., an average AUC score of 74.67% and 74.96% for the non-802.11 and 802.11 feature sets, respectively. Interestingly, this result is different from that of shallow classification: while in both cases the best results were obtained with the 802.11 feature set, the divergence between the top performer in terms of AUC score is almost 15% and 0.9% for shallow classification and DNN analysis, respectively. It is left for future work to investigate whether more advanced DNN models, including time-series-based anomaly detection, can significantly augment the detection scores. Further, as expected, between the two DNN approaches, the MLP model was clearly the fastest in terms of total execution time, independently of the utilized set. Figure 2 and Figure 3 depict the accuracy and validation performance of loss per epoch per feature set. As observed in Figure 2, for certain epochs, there exist several fluctuations in the validation loss of the 802.11 set; nevertheless, the maximum difference stays at ≈0.1%. Recall that validation loss values above the train loss curve may be an indication of overfitting.

**Figure 4:**
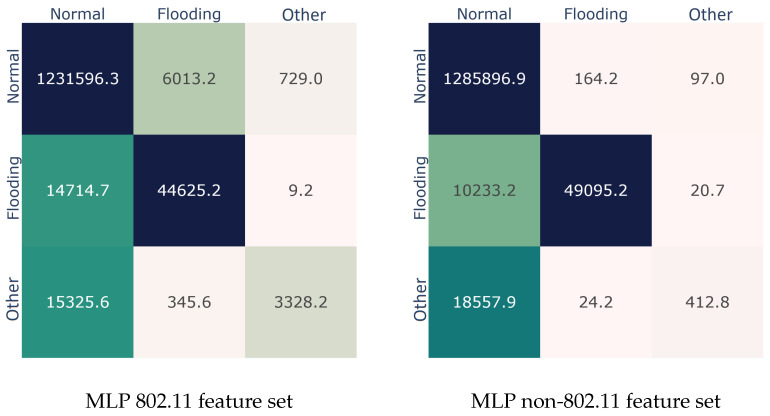
Best performer (MLP) confusion matrices per feature set.

To draw a clearer picture of the results, Figure 4 illustrates the confusion matrices for both feature sets. As with Figure 1 regarding the Bagging confusion matrices, the non-802.11 set performed better on the *Normal* class, missed about 17.2% of the *Flooding* class, and completely misfired on the *Other* class. It is interesting that MLP performed poorly with the 802.11 set of features too. For instance, it misclassified 26.9% samples of the *Flooding* class and 82.3% samples of the *Other* class. Only the *Normal* class presents an equivalent prediction rate to that of bagging in Section 4.1.

## 5. Delving into Feature Analysis

This section elaborates on the selected features. First off, we examine the importance of each feature on both feature sets. Second, we construct an artificial feature that could potentially assist in predicting the most challenging class, namely *Other*. Thirdly, and more interestingly, we investigate if using the two feature sets in tandem can increase the prediction rate of an ML model. For each of the aforementioned cases, only the best ML performers, i.e., LightGBM and Bagging were considered.

### 5.1. Feature Importance

Feature importance aims at inferring the dominant features, i.e., those which possibly bear the greater information for the ML model. To this end, a permutation importance analysis was carried out using LightGBM. The analysis used 10% of the stratified data from each feature set. Precisely, LightGBM was trained with a 10% subset of stratified samples and tested with a different 10% subset of stratified samples.

**Figure 5:**
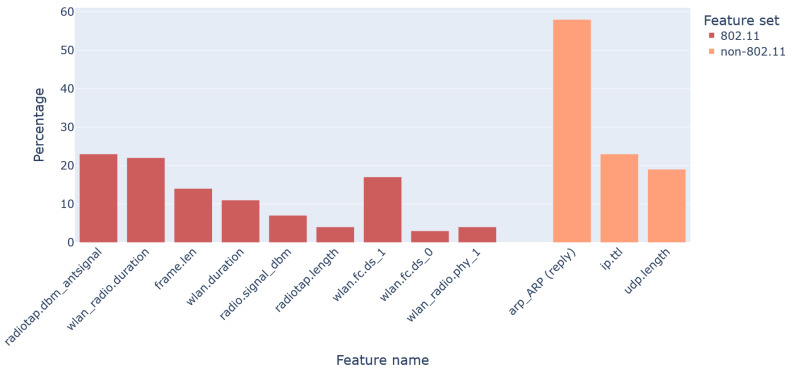
Feature importance through LightGBM for both the feature sets. All the insignificant features for both the feature sets were removed.

As illustrated in Figure 5, the analysis of the 802.11 features showed that six of them offer the most information: *frame.len*, *radiotap.dbm_antsignal*, *radiotap.length*, *wlan.duration*, *wlan_radio.duration*, and *wlan_radio.signal_dbm*. Further, the same type of analysis on the non-802.11 features revealed that only three of them, namely *arp*, *ip.ttl*, and *udp.length*, provide significant information. This is a logical result, since ARP and UDP features were more important for the *Flooding* class, while the TCP ones were assisted mostly in the detection of the *Other* class. On the flip side, this result also entails that the rest of the non-802.11 features have almost zero contribution, especially in the detection of the *Other* class, which is by far the most challenging. It is to be noted that while a couple of 802.11 features, namely *wlan.fc.ds* and *wlan_radio.phy* did have some importance, they were not picked because the useful information did not pertain to the feature as a whole, but to specific columns, say, *wlan.fc.ds_1*, due to the use of the OHE technique. As a result, if used, such a feature may introduce more noise rather than improve the detection capacity of the algorithm.

After dropping the insignificant features per set, we repeated the experiments, and the results for the two best performers are summarized in Table 7. Once more, the Bagging model was superior, yielding an AUC score of 90.71% on the 802.11 reduced set. This result corroborates the feature importance analysis; indeed, the dropped features do not contain useful information, since, vis-á-vis the results of Table 4, in terms of the AUC metric, Bagging lost only 0.05% and 0.71% for the 802.11 and non-802.11 feature sets, respectively. On the negative side, the reduced non-802.11 feature set completely missed the *Other* class, classifying its instances as *Normal* ones. Nevertheless, it managed to identify the *Normal* class with great success, misplacing approximately 100 samples in each fold.

The above-mentioned results on feature importance corroborate pretty well the outcomes of both kinds of analysis given in Section 4.1 and Section 4.2, and the current one: the 802.11 feature set produces better results vis-à-vis the non-802.11 set, and this out turn is far more obvious when it comes to shallow analysis. With reference to Figure 5, this result can be mainly attributed to a couple of key factors. First, the non-802.11 set misses the 802.3 *frame.len* feature because AWID3 was created in an 802.11 set. Conversely, this important feature is included in the 802.11 feature set, contributing appreciably to the detection of attacks. Second, a quartet of features, namely *radiotap.dbm_antsignal*, *wlan_radio.duration*, *wlan_duration*, and *radio_signal_dbm*, incorporated in the 802.11 set evidently aid in pinpointing the attacker, while the non-802.11 set misses this information. The reader should however keep in mind that these observations and findings are closely tied to the feature sets of Table 2. That is, the possible refinement and expansion of the feature sets depending on the particular case are left for future work.

### 5.2. Conflating the Feature Sets

**Figure 6:**
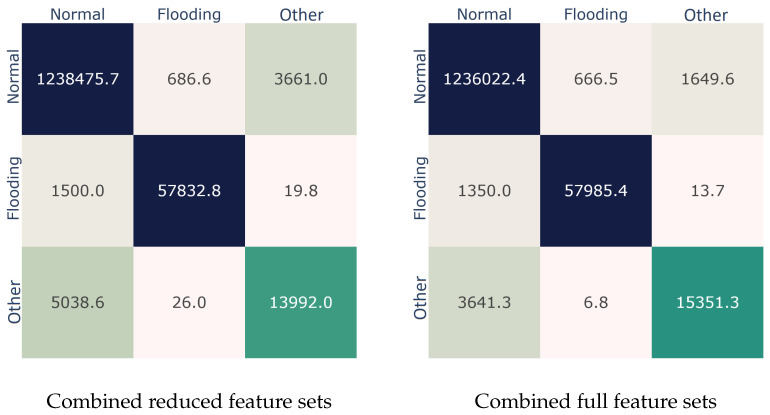
Best performer (bagging) confusion matrices considering the analysis of either reduced set (**left**), or full set (**right**) features.

In light of the analysis in Section 4.1, Section 4.2 and Section 5.1, an important question emerges: what if both these feature sets are exploited in tandem? To provide an answer, as shown in Table 8, we examined the detection performance of both the full and the reduced feature sets when used jointly. Simply put, the combined full feature set includes all the 33 features of Table 2, while the combined reduced set comprises the nine features depicted in Figure 5. As observed from Table 8, the combined feature set produces substantially better results in comparison to the case each set is used separately. Precisely, the gain in terms of the AUC metric is +4.52% over that of the full 802.11 16-features set given in Table 4. A significant AUC improvement (almost 3%) is also perceived in the percentage of the combined reduced set vis-à-vis that is seen in Table 7 regarding the 802.11 reduced feature set. Figure 6 elaborates on this outcome by illustrating the respective confusion matrices. As observed, in comparison to the results of Section 4.1, the combined feature set improved the prediction rate of the model by about 0.32%, 2.07%, and 18.42%, for the *Normal*, *Flooding*, and *Other* classes, respectively.

### 5.3. Use of Engineered Features

While the combined full feature set did augment the AUC score up to almost 95.30%, it would be interesting to indicatively examine the potential of (mostly empirically-derived) engineered features in possibly ameliorating this score. This demonstrative effort would also serve as a reference and guidance for future work. Obviously, based on the preceding discussions, the most cumbersome to detect class is the *Other*. To this end, for the explanation given below, we consider an enterprise network, that is, a similar setting to that deployed in the creation of AWID3. In such a network realm, the chance of a client machine being (also) utilized as a server is practically tiny. Nevertheless, such strange behavior, i.e., a local machine to serve a dual role, is exhibited in the *botnet* attack contained in AWID3. That is, the opponent, an insider in this case, operates a Command and Control (C2) server to herd and manage infected hosts (bots) in the local network. Based on this observation, using the same dataset, i.e., the *Botnet* pcap file, we used the pseudocode of Algorithm  1 for constructing an artificial feature dubbed “Insider”.

Specifically, this feature concentrates on the local IP address of each client. Namely, when the packets are sent from one client machine to another (client-to-client), the respective traffic samples were flagged with 1, otherwise (client-to-server) with 0. After its generation, the feature was preprocessed with OHE. It is argued that this engineered feature does not affect the generalization of the produced ML models, since it does not directly rely on the IP addresses per se, but only considers the correlation between them (client-to-client). In other words, a client’s IP address can change for different reasons, say, Dynamic Host Configuration Protocol (DHCP), alterations in network topology, and so on, but the model will be trained to detect weird communication patterns between local network nodes having a client role. Obviously, as with all the other features, this one is constructed based on readily available (not typically encrypted) packet-level information.

**Figure 7:**
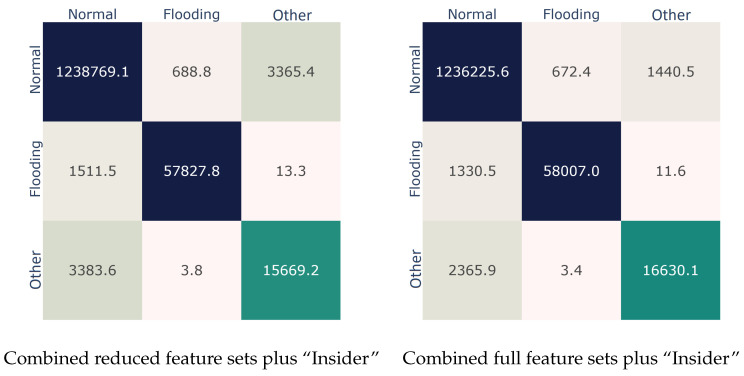
Best performer (Bagging) confusion matrices considering the engineered feature.

To assess the contribution of this feature to the detection performance, we utilized it alongside a triad of feature sets: the reduced 802.11 one, the reduced combined one, and the combined full set. The results per examined set are given in Table 9. Obviously, in all three cases, the engineered feature favorably improved the AUC score in the range of 2% to 3%. For instance, regarding the combined full feature set, the detection performance increased by nearly 1.5%. Looking at the confusion matrices presented in Figure 7, this betterment is clearly due to the improved identification of the *Other* class. Precisely, as expected, the addition of this single feature rendered possible the detection of the samples belonging to the *botnet* attack: in comparison to the two confusion matrices in Figure 7, the algorithm (bagging) was now able to correctly classify much more (around +6.7%) samples of the *Other* class, which in Figure 6 were misclassified in the *Normal* class.
**Algorithm 1** Algorithm for constructing the “Insider” feature.**Require:**list_of_clients_ip**Require:**data    **for**
i=0; i< length(list_of_clients_ip); i++
**do**          src←data[i][ip.src]          dst←data[i][ip.dst]          counter_src←0          counter_dst←0          **for** j=0; j< length(list_of_clients_ip); j++ **do**                tmp←list_of_clients[j]                **if** src is in tmp **then**                      counter_src←1                **else if** dst is in tmp **then**                      counter_dst←1                **else if** counter_src and counter_dst == 1 **then**                      data[i][insider]←1                      break                **end if**          **end for**          **if** counter_src == 0 or counter_dst == 0 **then**                data[i][insider]←0          **end if**    **end for**

## 6. Conclusions

Since its inception back in the late 1990s, Wi-Fi has ripened into a full-fledged mature technology being utilized in numerous everyday applications. Nevertheless, IEEE 802.11 networks are alluring to attackers as well. That is, in absence of any intrinsic network access control as in its wired counterparts, an assailant can either attack the wireless network directly or used it as an (anonymous) springboard for assaulting other networks; the opponent can be anywhere in the vicinity or further afield depending on the strength/type of the wireless signal/equipment. Furthermore, while 802.11 security features have greatly evolved and enriched in the passing of time, new vulnerabilities emerge. In this context, an intriguing from an IDS viewpoint issue is to examine the potential of combining the information stemming from both wired and wireless protocols in such commonplace hybrid network realms, to possibly improve the detection performance. From that standpoint, the current study aspires to set the ground for IDS that hinge on diverse feeds in terms of network traffic features. Differently to the related work, the ultimate goal here is to investigate if and to what degree application layer attacks can be detected with lower layer features, either or both frame-level or packet-level, which however are readily accessible, meaning neither encrypted nor anonymized.

While this effort in the context of this paper is concentrated on IEEE 802.11 networks, future work may exploit the same methodology for other mainstream network access technologies, including cellular. In short, the analysis conducted in the above sections suggests that when features stemming from different network realms and layers of the protocol stack are used alongside each other, the detection performance of the ML model is increased. With reference to our experiments, this boost rose up to almost 95.3% in terms of the AUC metric, which is significantly greater (around 4.5%) vis-à-vis the best result obtained with just the 802.11 feature set. Finally, yet importantly, it was demonstrated that the inclusion of engineered, yet generalized enough, features grounded in empirical evidence and/or theoretical insight can improve the prediction capacity of the ML model; this can be particularly beneficial for detecting challenging attacks exhibiting a diminutive and imperceptible footprint. Nevertheless, a thorough investigation of this potential is well beyond the scope of this paper and is left for future work. Along with the previous future direction, a different one could aim at experimenting with diverse sets of non-802.11 cherry-picked features originating from diverse protocols in the protocol stack.

## Figures and Tables

**Table 1:** Set of features referenced in the related work. The rightmost column denotes the chosen ML methodology that was applied per feature set: Supervised/Unsupervised Swallow Classification (S/USC), Swallow Classification (SC), Genetic Programming (GP), Neural Networks (NN), Deep Neural Networks (DNN). The features in bold were also used in the context of this work, as explained further down in Section 3.

Features	Work	Method
arp.dst.proto_ipv4, **arp.proto.type**	[9,15]	NN, SC
arp.src.proto_ipv4, ipv6.dst		
ipv6.src, tcp.time_delta		
tcp.window_size_value		
**arp.hw.size**, **arp.opcode**	[21,23]	FL
**tcp.analysis**	[9,15]	S/USC
**tcp.analysis.retransmission**	[23,24]	S/ USC, DNN
tcp.dstport	[9,11,20]	NN, SC
tcp.flags	[9,11,13,15]	NN, SC, GP
tcp.srcport	[9,11]	NN, SC, DNN, GP
**tcp.checksum.status**	[11,13,15]	NN, S/ USC, DNN
**tcp.flags.ack**, **tcp.flags.fin**, **tcp.flags.push**	[9,11,13,15,19,20]	NN, S/ USC, DNN, GP
**tcp.flags.reset**, **tcp.flags.syn**		
tcp.len, tcp.nxtseq		
tcp.seq, tcp.window_size, udp.checksum		
**udp.length**	[9,11,13,15,19]	NN, S/ USC, DNN, GP
dns.count.queries, dns.flags.recdesired	[9,11,15,19,22,23,25,29]	NN, S/USC
dns.flags.response		
icmp.ident, icmp.seq, icmp.seq_le		
ip.checksum.status, ip.dst, ip.hdr_len		
ip.src, ip.version		
tcp.analysis.bytes_in_flight		
tcp.analysis.initial_rtt		
tcp.analysis.push_bytes_sent		
tcp.options.timestamp.tsval		
tcp.payload, tcp.stream		
tcp.time_relative, tcp.window_size_value		
tcp.window_size_scalefactor		
udp.checksum.status, udp.stream		
udp.dstport, udp.srcport	[9,11,15,19,20]	NN, S/USC
ip.flags, ip.flags.df, ip.len, ip.proto, **ip.ttl**	[9,11,13,15]	NN, S/USC, GP
udp.checksum_bad	[11,13,20]	SC, GP
udp.checksum_coverage		
udp.checksum_good		
tcp.flags.urg, tcp.hdr_len	[11,13,15,19]	SC, DNN, GP
tcp.checksum_good	[9,11,13]	SC, GP
tcp.dstport		
tcp.flags.cwr		
tcp.flags.ecn		
data.len	[15]	S/USC
smb.cmd	[11]	S/USC
ssl.handshake.ciphersuite		
ssl.handshake.version		
ip.checksum, ip.checksum_bad	[9,11,13]	S/USC, GP
ip.checksum_good, ip.flags.mf		
ip.flags.rb, ip.frag.offset		
tcp.ack, tcp.checksum		
tcp.checksum_bad		

**Table 2:** The selected sets of features. The 802.11 features were selected from [8]. The rightmost columns per subtable name the normalization step per feature followed during data preprocessing.

802.11 Features (16)		Non-802.11 Features (17)	
Feature name	Preprocessing	Feature name	Preprocessing
frame.len	Min-Max	udp.length	Min-Max
radiotap.dbm_antsignal	Min-Max	ip.ttl	Min-Max
radiotap.length	Min-Max	arp	OHE
wlan.duration	Min-Max	arp.proto.type	OHE
wlan_radio.duration	Min-Max	arp.hw.size	OHE
wlan_radio.signal_dbm	Min-Max	arp.proto.size	OHE
radiotap.present.tsft	OHE	arp.hw.type	OHE
wlan.fc.type	OHE	arp.opcode	OHE
wlan.fc.subtype	OHE	tcp.analysis	OHE
wlan.fc.ds	OHE	tcp.analysis.retransmission	OHE
wlan.fc.frag	OHE	tcp.option_len	OHE
wlan.fc.moredata	OHE	tcp.checksum.status	OHE
wlan.fc.protected	OHE	tcp.flags.ack	OHE
wlan.fc.pwrmgt	OHE	tcp.flags.fin	OHE
wlan.fc.retry	OHE	tcp.flags.push	OHE
wlan_radio.phy	OHE	tcp.flags.reset	OHE
		tcp.flags.syn	OHE

**Table 3:** Parameter values per classifier. A hyphen denotes that the specific hyperparameter is inapplicable to that classifier.

Parameters	DT	LightGBM	Bagging
max_depth	100	100	–
max_leaf_nodes	200	–	–
min_samples_leaf	2	–	–
ccp_alpha	1 × 10^−5^	–	–
max_bin	–	200	–
min_child_samples	–	30	–
min_data_in_bin	–	50	–
n_estimators	–	300	50
num_leaves	–	300	–
learning_rate	–	0.01	–
reg_alpha	–	0.1	–
reg_lambda	–	0.1	–
n_jobs	–	1	–
max_samples	–	–	500,000

**Table 4:** Results of swallow classification on each feature set. Best and worse AUC and F1 scores are shown in green or orange text, respectively. The term T.t. in the rightmost column stands for total execution time, i.e., training and testing.

Model Name	AUC	Prec.	Recall	F1	Acc	T.t.
*802.11 set*						
DT	88.98	90.96	83.19	86.34	98.59	00:45:33
LightGBM	90.19	91.55	84.95	87.68	98.71	11:44:16
Bagging	90.77	91.08	85.85	88.07	98.73	03:06:34
*Non-802.11 set*						
DT	76.21	91.38	63.45	66.02	98.08	00:35:26
LightGBM	76.24	91.94	63.48	66.02	98.08	09:31:52
Bagging	76.28	91.70	63.51	66.02	98.08	03:43:17

**Table 5:** Parameter values for the DNN algorithms. A value of “/3” or “/2” in the MLP Dropout parameter indicates the number of layers in which this parameter had the designated value. The layer values are calculated without including the input and output ones. A hyphen defines an irrelevant option for this DNN model. SCC stands for the Sparse Categorical Crossentropy.

Parameters	MLP	Autoencoders
Activator	ReLU	ReLU
Output activator	Softmax	Softmax
Initializer	He_uniform	–
Optimizer	SGD	SGD
Momentum	0.9	0.9
Dropout	0.25/3–0.2/2	0.25
Learning rate	0.01	0.01
Loss	SCC	SCC
Batch Norm.	Yes	Yes
Hidden layers	5	7
Nodes (Per layer)	100/80/60/40/20	80/60/40/20/40/60/80
Batch size	170	

**Table 6:** Results for DNN models. Best and worse AUC and F1 scores are shown in green or orange text, respectively.

Model Name	AUC	Prec.	Recall	F1	Acc	Epochs	T.t.
*802.11 set*							
MLP	75.53	89.29	64.05	69.40	97.17	16.3	50:02:30
AE	74.96	88.93	63.29	68.50	97.05	22	69:12:58
*Non-802.11 set*							
MLP	74.67	92.56	61.92	64.48	97.86	5	13:08:51
AE	74.67	92.67	61.61	64.49	97.86	22.7	73:39:31

**Table 7:** Analysis on the reduced set of features. Best and worse AUC and F1 scores are shown in green or orange text, respectively.

Model Name	AUC	Prec.	Recall	F1	Acc	T.t.
*Reduced 802.11 set: 6 features*						
LightGMB	89.55	91.79	83.95	87.08	98.68	07:51:47
Bagging	90.71	90.56	85.79	87.84	98.70	00:44:25
*Reduced non-802.11 set: 3 features*						
LightGMB	75.57	94.95	62.44	64.05	98.04	04:41:38
Bagging	75.57	94.95	62.44	64.05	98.04	00:47:46

**Table 8:** Results on the combined feature sets. Best and worse AUC and F1 scores are shown in green or orange text, respectively.

Model Name	AUC	Prec.	Recall	F1	Acc	T.t.
*Combined reduced sets: 6+3 features*						
LightGMB	92.86	93.41	88.91	90.99	99.17	07:45:01
Bagging	93.63	92.47	90.17	91.28	99.17	01:34:41
*Combined full sets: 16+17 features*						
LightGMB	95.20	96.59	92.61	94.48	99.45	06:39:17
Bagging	95.29	96.23	92.77	94.40	99.44	05:28:02

**Table 9:** Results on feature sets embracing the “Insider” engineered feature. Best and worse AUC and F1 scores are shown in green or orange text, respectively.

Model Name	AUC	Prec.	Recall	F1	Acc	T.t.
*Reduced 802.11 feature set (6) plus the engineered feature*						
LightGMB	92.54	93.46	88.76	90.83	98.92	07:20:22
Bagging	93.46	92.34	90.22	91.20	98.93	01:00:51
*Combined reduced feature sets (6+3) plus the engineered feature*						
LightGMB	94.74	94.45	91.93	93.15	99.32	07:35:55
Bagging	95.46	93.56	93.10	93.33	99.32	01:43:42
*Combined full feature sets (16+17) plus the engineered feature*						
LightGMB	96.56	97.25	94.81	95.99	99.56	08:08:13
Bagging	96.70	96.84	95.03	95.91	99.55	06:43:37

## Data Availability

The AWID3-CSV dataset is available for download at https://icsdweb.aegean.gr/awid/download-dataset (accessed on 24 March 2022).

## Abbreviations

The following abbreviations are used in this manuscript:

Acc	Accuracy
ANN	Artificial Neural Networks
ARP	Address Resolution Protocol
AUC	Area Under ROC Curve
AWID3	Aegean Wi-Fi Intrusion Dataset
C2	Command and Control
CNN	Convolutional Neural Network
CSV	Comma-Separated Values
DDoS	Distributed Denial of Service
DHCP	Dynamic Host Configuration Protocol
DNN	Deep Neural Networks
DNS	Domain Name Service
DoS	Denial of Service
DT	Decision Tree Algorithm
FNN	Feedforward Neural Networks
FPR	False Positive Rate
GP	Genetic Programming
GRU	Gated Recurrent Unit
HTTP	Hypertext Transfer Protocol Secure
IDS	Intrusion Detection Systems
IP	Internet Protocol
KNN	K-Nearest Neighbors
LSTM	Long Short-Term Memory Algorithm
MiTM	Man in The Middle
ML	Machine Learning
MLP	Multilayer Perceptron
MNB	Multinomial Naïve Bayes
NN	Neural Networks
OHE	One-Hot Encoding
PMF	Protected Management Frames
Prec	Precision
ROC Curve	Receiver Operating Characteristic Curve
RF	Random Forest Algorithm
RNN	Recurrent Neural Network
S/USC	Supervised/Unsupervised Swallow Classification
SAE	Simultaneous Authentication of Equals
SBB	Symbiotic Bid-based
SC	Swallow Classification
SCC	Sparse Categorical Crossentropy
SGD	Stochastic Gradient Descent
SSDP	Simple Service Discovery Protocol
SSH	Secure Shell
SVM	Support Vector Machine
TCP	Transmission Control Protocol
UDP	User Datagram Protocol
VoWiFi	Voice over Wi-Fi
Wi-Fi	Wireless Fidelity
WIDS	Wireless Intrusion Detection Systems

## References

[1] Chatzoglou E., Kambourakis G., Kolias C. (2022). Your WAP Is at Risk: A Vulnerability Analysis on Wireless Access Point Web-Based Management Interfaces. Secur. Commun. Netw..

[2] Chatzoglou E., Kambourakis G., Kolias C. (2021). Empirical Evaluation of Attacks Against IEEE 802.11 Enterprise Networks: The AWID3 Dataset. IEEE Access.

[3] Chatzoglou E., Kambourakis G., Kolias C. (2022). How is your Wi-Fi connection today? DoS attacks on WPA3-SAE. J. Inf. Secur. Appl..

[4] Vanhoef M., Ronen E. Dragonblood: Analyzing the Dragonfly Handshake of WPA3 and EAP-pwd. Proceedings of the 2020 IEEE Symposium on Security and Privacy (SP).

[5] Tripathi N., Hubballi N. (2021). Application Layer Denial-of-Service Attacks and Defense Mechanisms: A Survey. ACM Comput. Surv..

[6] Xie Y., Yu S. (2009). Monitoring the application-layer DDoS attacks for popular websites. IEEE/ACM Trans. Netw..

[7] Srivatsa M., Iyengar A., Yin J., Liu L. (2008). Mitigating application-level denial of service attacks on Web servers: A client-transparent approach. ACM Trans. Web.

[8] Chatzoglou E., Kambourakis G., Kolias C., Smiliotopoulos C. (2022). Pick quality over quantity: Expert feature selection and data preprocessing for 802.11 Intrusion Detection Systems. IEEE Access.

[9] Ge M., Fu X., Syed N., Baig Z., Teo G., Robles-Kelly A. Deep Learning-Based Intrusion Detection for IoT Networks. Proceedings of the 2019 IEEE 24th Pacific Rim International Symposium on Dependable Computing (PRDC).

[10] Koroniotis N., Moustafa N., Sitnikova E., Turnbull B. (2019). Towards the development of realistic botnet dataset in the Internet of Things for network forensic analytics: Bot-IoT dataset. Future Gener. Comput. Syst..

[11] Alsirhani A., Sampalli S., Bodorik P. DDoS Detection System: Utilizing Gradient Boosting Algorithm and Apache Spark. Proceedings of the 2018 IEEE Canadian Conference on Electrical Computer Engineering (CCECE).

[12] Anonymized Internet Traces 2015. https://catalog.caida.org/details/dataset/passive_2015_pcap.

[13] Alshammari R., Zincir-Heywood A.N. (2011). Can encrypted traffic be identified without port numbers, IP addresses and payload inspection?. Comput. Netw..

[14] Vinayakumar R., Soman K., Poornachandran P. Secure shell (ssh) traffic analysis with flow based features using shallow and deep networks. Proceedings of the 2017 International Conference on Advances in Computing, Communications and Informatics (ICACCI).

[15] Li T., Hong Z., Yu L. Machine Learning-based Intrusion Detection for IoT Devices in Smart Home. Proceedings of the 2020 IEEE 16th International Conference on Control Automation (ICCA).

[16] Haddadi F., Runkel D., Zincir-Heywood A.N., Heywood M.I. (2014). On Botnet Behaviour Analysis Using GP and C4.5.

[17] Yang Y., Kang C., Gou G., Li Z., Xiong G. TLS/SSL Encrypted Traffic Classification with Autoencoder and Convolutional Neural Network. Proceedings of the 2018 IEEE 20th International Conference on High Performance Computing and Communications; IEEE 16th International Conference on Smart City; IEEE 4th International Conference on Data Science and Systems (HPCC/SmartCity/DSS).

[18] Su M.Y. (2011). Real-time anomaly detection systems for Denial-of-Service attacks by weighted k-nearest-neighbor classifiers. Expert Syst. Appl..

[19] Yuan X., Li C., Li X. DeepDefense: Identifying DDoS Attack via Deep Learning. Proceedings of the 2017 IEEE International Conference on Smart Computing (SMARTCOMP).

[20] Balkanli E., Zincir-Heywood A.N., Heywood M.I. Feature selection for robust backscatter DDoS detection. Proceedings of the 2015 IEEE 40th Local Computer Networks Conference Workshops (LCN Workshops).

[21] Ferrag M.A., Friha O., Hamouda D., Maglaras L., Janicke H. (2022). Edge-IIoTset: A New Comprehensive Realistic Cyber Security Dataset of IoT and IIoT Applications for Centralized and Federated Learning. IEEE Access.

[22] Ge M., Syed N.F., Fu X., Baig Z., Robles-Kelly A. (2021). Towards a deep learning-driven intrusion detection approach for Internet of Things. Comput. Netw..

[23] Al-Daweri M.S., Abdullah S., Ariffin K.A.Z. (2021). An adaptive method and a new dataset, UKM-IDS20, for the network intrusion detection system. Comput. Commun..

[24] Schneider P., Böttinger K. (2018). High-Performance Unsupervised Anomaly Detection for Cyber-Physical System Networks. Proceedings of the 2018 Workshop on Cyber-Physical Systems Security and PrivaCy.

[25] Fruehwirt P., Schrittwieser S., Weippl E. Using machine learning techniques for traffic classification and preliminary surveying of an attackers profile. Proceedings of the Talk: ASE International Conference on Privacy, Security, Risk and Trust (PASSAT).

[26] Liu H., Lang B. (2019). Machine Learning and Deep Learning Methods for Intrusion Detection Systems: A Survey. Appl. Sci..

[27] Kalimuthan C., Arokia Renjit J. (2020). Review on intrusion detection using feature selection with machine learning techniques. Mater. Today Proc..

[28] Saranya T., Sridevi S., Deisy C., Chung T.D., Khan M. (2020). Performance Analysis of Machine Learning Algorithms in Intrusion Detection System: A Review. Procedia Comput. Sci..

[29] Huang W., Peng X., Shi Z., Ma Y. Adversarial attack against LSTM-based DDoS intrusion detection system. Proceedings of the 2020 IEEE 32nd International Conference on Tools with Artificial Intelligence (ICTAI).

[30] Kampourakis V., Kambourakis G., Chatzoglou E., Zaroliagis C. (2022). Revisiting man-in-the-middle attacks against HTTPS. Netw. Secur..

[31] Chatzoglou E., Kouliaridis V., Karopoulos G., Kambourakis G. (2022). Revisiting QUIC attacks: A comprehensive review on QUIC security and a hands-on study. Res. Sq. Prepr..

[32] Kambourakis G., Karopoulos G. (2022). Encrypted DNS: The good, the bad and the moot. Comput. Fraud. Secur..

